# Enhancing Crop Resilience: Insights from Labdane-Related Diterpenoid Phytoalexin Research in Rice (*Oryza sativa* L.)

**DOI:** 10.3390/cimb46090634

**Published:** 2024-09-23

**Authors:** Shiquan Bian, Zhong Li, Shaojie Song, Xiao Zhang, Jintao Shang, Wanli Wang, Dewen Zhang, Dahu Ni

**Affiliations:** 1 Key Laboratory of Rice Germplasm Innovation and Molecular Improvement of Anhui Province, Rice Research Institute, Anhui Academy of Agricultural Sciences, Hefei 230031, China; 2 Agricultural Technology Extension Center of Linping District, Hangzhou 311199, China

**Keywords:** rice, labdane, diterpenoid, phytoalexins, resilience

## Abstract

Rice (*Oryza sativa* L.), as one of the most significant food crops worldwide, holds paramount importance for global food security. Throughout its extensive evolutionary journey, rice has evolved a diverse array of defense mechanisms to fend off pest and disease infestations. Notably, labdane-related diterpenoid phytoalexins play a crucial role in aiding rice in its response to both biotic and abiotic stresses. This article provides a comprehensive review of the research advancements pertaining to the chemical structures, biological activities, and biosynthetic pathways, as well as the molecular regulatory mechanisms, underlying labdane-related diterpenoid phytoalexins discovered in rice. This insight into the molecular regulation of labdane-related diterpenoid phytoalexin biosynthesis offers valuable perspectives for future research aimed at improving crop resilience and productivity.

## 1. Introduction

As the global population steadily grows, the demand for food escalates accordingly, necessitating greater efforts to ensure sustainable agricultural production and food security worldwide [1,2]. As an important member of the Gramineae family, rice (*Oryza sativa* L.) is one of the most crucial food crops in the world, serving as the staple food for half of the global population, with particular significance in East and Southeast Asian countries [3,4,5]. Thus, the stable production of rice is directly related to global food security and the livelihoods of hundreds of millions of people.

However, as the severe natural climatic conditions continue to worsen, the situation with pests and diseases has also intensified. These unfavorable factors not only increase the uncertainty in the growth process of rice but also make the prevention and control of pests and diseases more complex and difficult, posing a serious threat to rice yield and quality [6,7,8,9]. Therefore, conducting research on rice resistance and cultivating new rice varieties with strong stress tolerance have become urgent requirements for ensuring global food security. This not only helps reduce food production losses caused by natural disasters and pests and diseases but also decreases reliance on chemical pesticides and fertilizers, thereby promoting the sustainable development of agricultural production [10,11,12].

In recent years, with the rapid development of molecular biology and biotechnology, scientists have gained a deeper understanding of the resistance mechanisms in rice. Among them, the research of phytoalexins in rice is particularly notable. These compounds play an important role in the resistance of rice to pests, diseases, and stress, providing new ideas and methods to improve the resistance of crops [13,14,15].

Phytoalexins are low-molecular-weight, secondary metabolites produced by plants in response to pathogen attack or other stress stimuli that play an essential role in plant defense mechanisms by inhibiting the growth and proliferation of pathogens, thereby reducing disease severity and enhancing plant resilience [16,17,18,19]. Phytoalexins display a diverse array of chemical structures, encompassing terpenes, phenols, flavonoids, and stilbenes, among others. Their biosynthesis is typically triggered in a localized and swift response following the recognition of a pathogen attack [20,21,22,23]. Within this field, the unique biological activities of labdane-related diterpenoid phytoalexins have garnered significant attention in rice resistance research due to their potential to confer targeted and durable resistance against a broad spectrum of pathogens [24,25].

Labdane-related diterpenoid phytoalexins originate from the labdane skeleton, a bicyclic diterpenoid precursor, via a sequence of intricate biosynthetic pathways. These compounds are pivotal in plant innate immunity, functioning as potent toxins or inhibitors that combat invading pathogens, including bacteria, fungi, and viruses [24,25]. The structural complexity of labdane-derived diterpenoids allows for a wide range of bioactivities, making them valuable not only in plant defense but also as potential leads for the development of novel agrochemicals and pharmaceuticals.

This review summarizes the current insights gained from research on labdane-related diterpenoid phytoalexins in rice, highlighting their biosynthesis pathways and key enzymes involved. Furthermore, this review envisions a promising future for leveraging the profound insights gained into labdane-related diterpenoid phytoalexins to devise innovative crop improvement strategies. These strategies, aimed at optimizing the biosynthetic pathways and enhancing the expression of key enzymes, hold the key to developing rice varieties with enhanced innate immunity and resilience. Such advancements, in turn, are poised to contribute substantially to the sustainability of agricultural systems worldwide, ensuring food security for growing populations amidst the challenges posed by climate change and resource constraints. Ultimately, this research endeavor represents a crucial step towards harnessing the full potential of plant natural products for sustainable agriculture and a more resilient global food system.

## 2. Chemical and Biological Activities of Rice Labdane-Related Diterpene Phytoalexins

### 2.1. Types and Structural Characteristics

**Figure 1:**
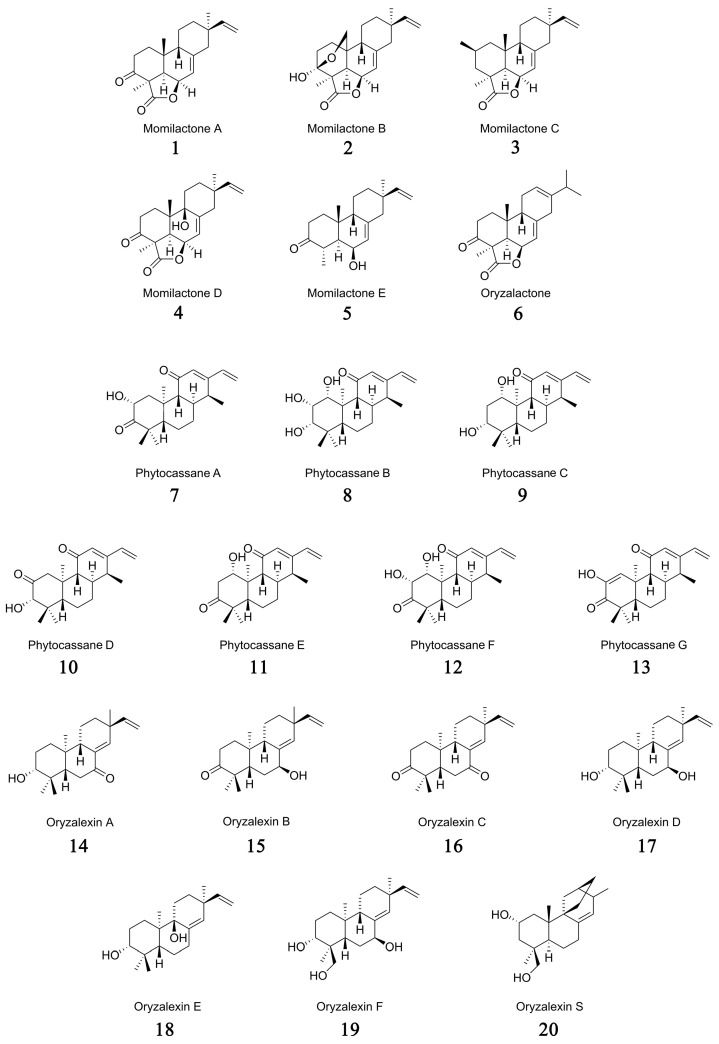
Chemical structures of labdane-type diterpene phytoalexins in rice.

Based on the distinct structures of their hydrocarbon precursors, these phytoalexins in rice can be categorized into three major groups: momilactones, phytocassanes, and oryzalexins (Figure 1). Specifically, the momilactones group encompasses momilactones A–E (**1**–**5**), oryzalactone (**6**) [26,27,28,29,30,31]; the phytocassanes group comprises phytocassanes A–G (**7**–**13**) [31,32,33,34]; and the oryzalexins group includes oryzalexins A–F (**14**–**19**) [35,36,37,38,39,40,41] and oryzalexin S (**20**) [34,42,43].

Structurally, momilactones belong to the 9β-H pimarane diterpenoids family, which has continuous chiral centers with a trans-*syn*-cis tricyclic skeleton [44,45,46]. Among them, momilactone A (**1**) and momilactone B (**2**) are the main components of labdane-type diterpene phytoalexins in rice, bearing a 19,6β-lactone structure [44,45,46]. These two important phytoalexins were initially isolated by Kato and colleagues from rice husks (*Oryza sativa* cv. Koshihikari) as growth inhibitors of lettuce (*Lactuca sativa*) seedlings [26,27]. In the subsequent 40 years, three more momilactone compounds were isolated and identified from rice blast infected leaves and named momilactones C (**3**), D (**4**), and E (**5**), as well as oryzalactone (**6**) [28,29,30,31]. With the development of research, more and more labdane-type diterpenoid phytoalexins have been identified in rice, named phytocassanes and oryzalexins, respectively [31,32,33,34,35,36,37,38,39,40,41,42,43].

Phytocassanes are a class of compounds featuring an ent-cassane diterpenoid skeleton with a ketone group at the C11 position [31,34]. Koga et al. isolated and identified four novel diterpenes with a cassane skeleton from the abundant phytoalexins produced in rice leaves infected by the rice blast fungus. These were named phytocassane A (**7**), phytocassane B (**8**), phytocassane C (**9**), and phytocassane D (**10**) [32]. Following this, phytocassane E (1β-hydroxy-12,15-cassadien-3,11-dione) (**11**) and phytocassane F (1α,2α-dihydroxy-ent-12,15-cassadiene-3,11-dione) (**12**) were isolated from rice cells cultured in suspension treated with extracts from the potato pathogen Phytophthora infestans hyphae and from rice leaves exposed to ultraviolet radiation, respectively [33,34]. As a didehydroisomer of phytocassane A (**7**), phytocassane G (**13**) was isolated and identified by Kariya et al. from the cultivated rice variety Basilanon [31].

Oryzalexins can be classified into two major groups according to their precursors. Oryzalexins A–F (**14**–**19**) belong to the ent-sandaracopimaradiene diterpene compounds derived from ent-sandaracopimara-8(14),15-diene, and oryzalexin S (**20**) belongs to the astemarane-like diterpene compounds derived from syn-stemar-13-ene via hydroxylation at C2a and C19 [34,35,36,37,38,39,40,41,42,43,44].

Oryzalexin A (3-hydroxy-7-oxo-sandaracopimaradiene) (**14**) was first isolated and identified from rice leaves infected with the rice blast fungus Pyricularia oryzae [35,36]. Subsequently, oryzalexin B (**15**) and oryzalexin C (**16**) were isolated and identified as two new antitoxins from rice leaves infected with the rice blast fungus Pyricularia oryzae by Akatsuka’s team in 1985 [37,38]. In 1986, Sekido et al. isolated a new phytoalexin, named oryzalexin D (**17**), from rice leaves infected with rice blast disease caused by Magnaporthe oryzae. The structure of oryzalexin D (**17**) was confirmed as 3,7-dihydroxy-(+)-sandaracopimaradiene through UV, mass spectrometry, nuclear magnetic resonance, and optical rotation methods. At 100 ppm, oryzalexin D (**17**) significantly inhibited the spore germination of Magnaporthe oryzae [39]. Kato et al. isolated oryzalexin E (**18**) and oryzalexin F (**19**) from rice leaves exposed to ultraviolet light and determined their structures and relative configurations as isopimara-8,(14),15-diene-3β,9α-diol and ent-isopimara-8(14),15-diene-3β,18-diol, respectively, through spectroscopic analysis [40,41]. Then, a novel antifungal phytoalexin, designated as oryzalexin S (**20**), from the rice leaves subjected to ultraviolet light exposure, was isolated and identified [42,43].

### 2.2. Biological Activities

#### 2.2.1. Antimicrobial

Previous studies have indicated that almost all of the labdane-type diterpene phytoalexins are isolated from leaves infected with blast fungus, suggesting that resistance to pathogens is the primary biological activity of these compounds [28,32,33,37,38,39,40,41,47,48,49,50].

Momilactone A (**1**) and momilactone B (**2**) exhibited potent inhibitory effects against the devastating rice blast fungus, *P. oryzae*, achieving 50% inhibition at concentrations as low as 5 μg/mL for momilactone A (**1**) and an even more impressive 1 μg/mL for momilactone B (**2**), thereby highlighting the exceptional efficacy of these compounds in combating this pathogenic threat [47]. What is more, momilactone A (**1**) and momilactone B (**2**) additionally demonstrated strong antifungal activity against *Cladosporium cucumerinum*, further underscoring their broad-spectrum inhibitory potential against fungal pathogens [28]. 

While momilactone A (**1**) and momilactone B (**2**) have been shown to possess significant inhibitory activity against various pathogens, their efficacy varies depending on the specific type of pathogen [48]. Fukuta et al.’s research underscores the superiority of momilactone B (**2**) over momilactone A (**1**) in terms of antibacterial activity, particularly against fungal pathogens such as *Botrytis cinerea* (IC_50_: 1.2 μg for momilactone B vs. 78.1 μg for momilactone A), *Fusarium solani* (IC_50_: 123.9 μg for momilactone B vs. 198.1 μg for momilactone A), and *Colletotrichum gloeosporioides* (IC_50_: 53.4 μg for momilactone B vs. 95.3 μg for momilactone A). Furthermore, momilactone B (**2**) displays a more pronounced inhibitory effect against bacterial species like *Pseudomonas ovalis*, *Bacillus cereus*, and *Bacillus pumilus* in comparison with momilactone A (**1**). However, when tested against *Escherichia coli*, both compounds exhibit comparable levels of inhibitory activity [48].

The research conducted by Koga et al. reveals that phytocassanes exhibited strong antifungal activity against M. grisea and R. solani, with phytocassanes A, B, C, and D (**7**–**10**) having ED_50_ values of 20, 4, 7, and 25 μg/mL, respectively, in preventing spore germination of *M. grisea* [32]. The research team led by Koga made a discovery in the form of phytocassanes E (**11**), which they identified in rice suspension-cultured cells infected with *Phytophthora infestans*. Their findings indicated that phytocassane E (**11**) possesses remarkable antifungal properties against *Magnaporthe grisea*. Specifically, the ED_50_ values for inhibiting spore germination and germ tube growth of this fungus were astonishingly low, at 6 and 2 μg/mL, respectively. Moreover, they pinpointed the hydroxyl group located at the C1 position as the primary functional group responsible for the exceptionally high antifungal activity exhibited by phytocassane E (**11**), shedding light on the molecular mechanisms underlying its potent effects [33].

Oryzalexins, a class of naturally occurring antimicrobial compounds in rice plants, contribute significantly to the plant’s innate defense mechanisms. Previous studies have shown that oryzalexins inhibit the growth of various fungal species, including those responsible for rice diseases like *Pyricularia oryzae* and brown spot (*Bipolaris oryzae*) [37,38,39,49,50]. When tested against *P. oryzae* spore germination, oryzalexins E (**18**) and F (**19**) exhibited ED_50_ values of 62.5 and 103 ppm, respectively [40,41].

#### 2.2.2. Nematode Resistance

In a study exploring the multifaceted roles of rice diterpenoid phytoalexins (DPs), researchers found that these well-known secondary metabolites, known for their resistance against foliar pathogens, are also produced and released by rice roots. They hypothesized that DPs may play a significant role in plant-nematode interactions, particularly in defending rice plants against phytoparasitic nematodes.

To test this hypothesis, they conducted transcriptome analysis on rice roots, focusing on the effects of infection by the root-knot nematode, Meloidogyne graminicola, and treatment with resistance-inducing chemicals on DP biosynthesis genes. They also evaluated the vulnerability of mutant rice lines with compromised DP biosynthesis to *M. graminicola*. Additionally, the study involved growing these mutants and their wild-type counterparts in field soil and utilizing metabarcoding to assess the influence of impaired DP biosynthesis on rhizosphere and root nematode communities [51].

The results reveal that *M. graminicola* suppresses DP biosynthesis genes early during its invasion, while resistance-inducing stimuli temporarily boost DP biosynthesis. Notably, the absence of DPs increased the rice plants’ susceptibility to *M. graminicola*. Metabarcoding analysis of wild-type and DP-deficient plants grown in field soil demonstrated that DPs significantly reshape the composition of rhizosphere and root nematode communities. In conclusion, diterpenoid phytoalexins are crucial players in both constitutive and inducible defense mechanisms against nematode pathogens in rice, and they play a pivotal role in structuring rice-associated nematode communities [51].

#### 2.2.3. Allelopathic

Numerous studies have conclusively demonstrated that momilactone A (**1**) and momilactone B (**2**) are the primary allelochemicals produced by rice. These compounds serve as potent natural weapons, effectively suppressing the growth of weeds [50,51,52]. These compounds, momilactone A (**1**) and momilactone B (**2**), play a crucial role in rice’s competitive ability within its ecological niche. They ensure rice’s dominance and healthy growth amidst the surrounding vegetation [52,53,54,55]. These intricate compounds, momilactone A (**1**) and momilactone B (**2**), are meticulously synthesized and sequestered within the roots of rice seedlings. They are strategically deployed into the rhizosphere through root exudates, gradually accumulating in the soil. Their presence acts as a formidable barrier, effectively inhibiting the seed germination and overall growth of neighboring weeds, such as barnyard grass and plantain. By disrupting seed germination processes, impeding root elongation, and stunting shoot growth, these allelochemicals ensure that rice thrives in its environment, unencumbered by competing vegetation [56,57,58]. The exact mechanisms by which momilactone A (**1**) and momilactone B (**2**) inhibit weed growth are complex. They may involve disrupting hormonal balance, inhibiting enzyme activity, or interfering with nutrient uptake. 

The research conducted by Kato-Noguchi and his team shows that the growth of seedlings of mustard greens (*Lepidium sativum* L.) and lettuce (*Lactuca sativa* L.) was significantly inhibited when exposed to momilactone B (**2**) solutions. The growth of mustard greens was inhibited at concentrations greater than 3 µM, while lettuce growth was inhibited at concentrations greater than 30 µM [59]. Chung et al. found that momilactones A (**1**) and momilactone B (**2**) also exhibited strong inhibitory activity against duckweed (*Lemna paucicostata*). In further experiments, they showed that when the concentration of momilactone B (**2**) reached 20 ppm, it completely inhibited the germination of three weed species: Chinese sprangletop (*Leptochloa chinensis* L.), redroot pigweed (*Amaranthus retroflexus* L.), and spike rush (*Cyperus difformis* L.) [60]. Upon external application, momilactone A (**1**) and momilactone B (**2**) were found to have notable inhibitory effects on the growth of both shoots and roots of barnyard grass (*E. crus-galli*). The growth was inhibited at concentrations exceeding 30 mmol/L for momilactone A (**1**) and 1 mmol/L for momilactone B (**2**), respectively [52].

#### 2.2.4. Drought Resistance

Apart from their bioactivities, such as resistance against harmful pathogens and allelopathy, momilactones also play a crucial role in enhancing rice’s ability to withstand abiotic stresses. Xuan’s research team conducted an analysis of the content of momilactone A (**1**) and momilactone B (**2**) in 30 rice varieties from diverse origins, including hybrids of indica and japonica subspecies, as well as exotic, glutinous, local, upland, and drought-tolerant rice. They further compared the correlation between the content of these momilactones and various traits, such as salt tolerance, drought tolerance, weed resistance, total flavonoid content, total phenolic content, and antioxidant capacity. The results reveal a strong correlation between the content of momilactone A and drought tolerance, with a correlation coefficient of 0.65 [61]. Zhang et al. found that *cps2*, *cps4,* and *cps2×4* double mutants were more sensitive to drought stress due to reduced stomatal closure under drought conditions compared with the wild type. This suggests that labdane-related diterpenoid phytoalexins may act as a regulatory switch that triggers stomatal closure in rice, potentially reflecting the role of these openings in microbial entry [62].

**Figure 2:**
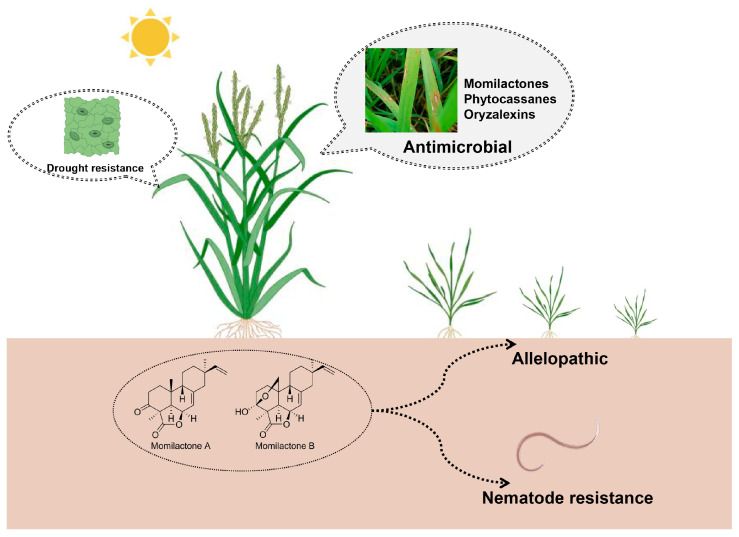
Biological activities of rice labdane-related diterpenoid phytoalexins.

Figure 2 displays the biological activities of rice labdane-related diterpenoid phytoalexins.

## 3. Biosynthesis of Rice Labdane-Related Diterpenoid Phytoalexins

Consistent with the biosynthetic pathways of other diterpenoids, the rice labdane-related diterpenoid phytoalexins (Momilactones, Phytocassanes, and Oryzalexins) all initiate from geranylgeranyl diphosphate (GGPP) as the starting material [63,64]. Initially, GGPP undergoes a series of enzymatic transformations catalyzed by a suite of diterpene synthases. These enzymes, each with its unique specificity and catalytic prowess, sculpt the GGPP backbone into intermediate structures that are progressively more complex and specialized. Concurrently, cytochrome P450 enzymes are renowned for their ability to perform oxidative modifications on a wide range of substrates, which intricately decorate the intermediate diterpenes with oxygen-containing functional groups, further diversifying their structures and enhancing their biological activities. This collaborative effort between diterpene synthases and cytochrome P450 enzymes results in the biosynthesis of momilactones, phytocassanes, and oryzalexins—a formidable trio of phytoalexins that arm rice plants against various biotic and abiotic stresses. 

### 3.1. Biosynthesis of Momilactones

A *syn*-CPS enzyme encoded by *OsCPS4* and located on chromosome 4 catalyzes the initial cyclization of the GGPP, converting into syn-copalyl diphosphate (*syn*-CPP) as a crucial precursor for momilactones [65]. Subsequently, the *syn*-pimara-7,15-diene synthase, encoded by *OsKSL4*, transforms *syn*-CPP into the momilactone tricyclic skeleton, (9β-H)-pimara-7,15-diene directly [66]. 

CYP99A2 and CYP99A3 occupy a central and crucial role within the intricate momilactone gene cluster, encoding cytochrome enzymes that meticulously orchestrate a vital oxygenation reaction, precisely targeting the C19 position of (9β-H)-pimara-7,15-diene. This enzymatic prowess deftly converts the intermediate into (9β-H)-Pimaradien-19-oic acid, a crucial and direct precursor that paves the way for the biosynthesis of both momilactone C (**3**) and momilactone E (**5**). Subsequently, (9β-H)-Pimaradien-19-oic acid undergoes further enzymatic modifications, elegantly transforming into 3β-hydroxy-(9β-H)-pimara-7,15-dien-19,6β-olide. This intricate and delicate pathway underscores the indispensable and paramount roles that CYP99A2 and CYP99A3 play in the synthesis of these biologically significant and impactful compounds [67].

Additionally, CYP76M8 and CYP701A8, located on the phytocassane gene cluster and associated with gibberellin biosynthesis, have also been shown to participate in momilactone biosynthesis. Research teams led by Kitaoka have elucidated the momilactone biosynthetic pathway and the evolutionary process of key enzymes, confirming the critical roles of CYP76M8 and CYP701A8 in momilactone biosynthesis. These enzymes directly catalyze the conversion of (9β-H)-pimara-7,15-diene into 3β-hydroxy-(9β-H)-pimara-7,15-dien-19,6β-olide [68]. 

Subsequently, 3β-hydroxy-(9β-H)-pimara-7,15-dien-19,6β-olide could either be directly converted into momilactone D (**4**) or, undergo complete oxidation catalyzed by a short-chain dehydrogenase/reductase (SDR) encoded by *OsMAS*, resulting in the formation of momilactone A (**1**) and momilactone B (**2**) [69,70].

### 3.2. Biosynthesis of Phytocassanes

Under the catalysis of the diterpene synthase CPS2 which is encoded by *OsCPS2*, GGPP first undergoes a cyclization reaction to form *ent*-CPP. Subsequently, KSL7 catalyzes the conversion of *ent*-CPP into *ent*-cassadiene [71,72,73]. Following this, the cytochrome P450 enzymes, specifically CYP76M7, CYP76M8 and CYP71Z7, engage in additional oxidative transformations of *ent*-Cassa-12,15-diene. More precisely, CYP76M7 and CYP76M8 are crucial in catalyzing the α-hydroxylation process at the C11 site of 3-hydroxy-cassadiene, whereas CYP71Z7 contributes to the hydroxylation reaction specifically at the C2 position within phytocassanes [74,75,76]. The sequential cytochrome-mediated hydroxylation modifications ultimately result in the transformation of *ent*-cassadiene into phytocassanes A–E (**7**–**12**).

### 3.3. Biosynthesis of Oryzalexins

In line with the biosynthetic pathway of the other two labdane-derived diterpenoid phytoalexins found in rice, the synthesis of oryzalexins A–F (**14**–**19**) begins with GGPP as the precursor. This initial substrate undergoes a catalytic transformation catalyzed by OsCPS4, resulting in the formation of *syn*-CPP [65]. Subsequently, OsKSL10 acts on *syn*-CPP, converting it into *ent*-Pimara-7,5-diene, thereby initiating the production of the specified oryzalexins [50]. 

Under the activity of CYP701A8, the *ent*-Pimara-7,5-diene undergoes a transformation, resulting in the formation of 3α-Hydroxy-ent-sandaracopimaradiene [77]. This intermediate serves as a crucial precursor in the biosynthesis of oryzalexins A-E (**14**–**18**), marking an essential step towards the production of these phytoalexins in rice. The P450 cytochrome enzymes, specifically CYP76M8 and CYP76M6, play pivotal roles in the hydroxylation process of the tricyclic precursor. CYP76M8 selectively hydroxylates the C-7β position, while CYP76M6 targets the C-9β position. These enzymatic modifications lead to the formation of 3α,7β-diol and 3α,9β-diol compounds, respectively, which are definitively identified as oryzalexin D (**17**) and oryzalexin E (**18**). This sequence of enzymatic reactions is crucial for the biosynthesis of these specific phytoalexins in rice [78]. Following the hydroxylation by CYP76M8 and CYP76M6, the structural modifications in oryzalexin D (**17**) proceed further with the oxidation of the 3α-hydroxy and accompanying 7β-hydroxy groups. This oxidation step is catalyzed by short-chain alcohol dehydrogenase/reductases encoded by *OsSDR110C-MI3* and *OsSDR110C-MS3*, producing oryzalexins A–C (**14**–**16**). This cascade of enzymatic activities in rice underscores the intricate and orchestrated biosynthesis of these phytoalexins [79].

After being synthesized by OsCPS4-catalyzed condensation of GGPP, the resulting *syn*-copalyl pyrophosphate (*syn*-CPP) undergoes a crucial transformation. This conversion is mediated by the *ent*-kaurene synthase enzyme, which is encoded by *OsKSL8* and catalyzes the cyclization of *syn*-CPP into *syn*-stemar-13-*ene*. This step marks a significant advance in the biosynthetic pathway of diterpenoid compounds in rice, leading to the formation of an intermediate that can further undergo diverse modifications to give rise to a variety of biologically active compounds [80,81]. 

In the subsequent biosynthetic step, the C19 position of *syn*-stemar-13-ene, the product of the *ent*-kaurene synthase reaction catalyzed by OsKLS8, undergoes hydroxylation. This hydroxylation is mediated by the cytochrome P450 enzymes CYP99A3 and its homolog CYP99A2, which specifically target the C19 carbon atom. The outcome of this enzymatic reaction is the formation of *syn*-stemar-13-en-19-ol, a pivotal precursor in the biosynthesis of oryzalexin S (**20**) [82]. This transformation represents a crucial branch point in the diterpenoid biosynthetic pathway, leading toward the production of bioactive compounds that are essential for rice defense mechanisms. In the final step of the biosynthetic pathway leading to oryzalexin S (**20**), the C2α position of *syn*-stemar-13-en-19-ol undergoes hydroxylation catalyzed by the cytochrome P450 enzymes CYP71Z21 and CYP71Z22 [82]. The result of this enzymatic reaction is the formation of oryzalexin S (**20**), a bioactive phytoalexin that plays a crucial role in the defense mechanisms of rice against various biotic and abiotic stresses. 

**Figure 3:**
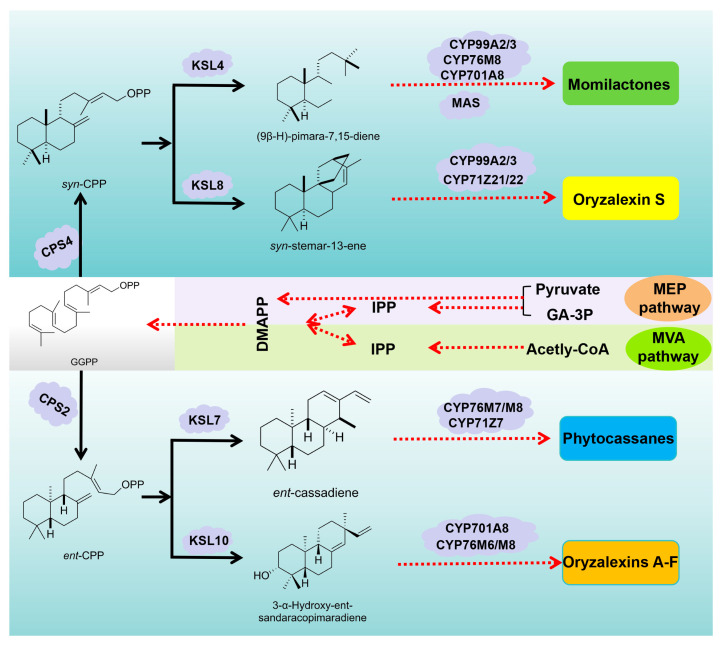
Biosynthetic pathways of rice labdane-related diterpenoid phytoalexins.

Biosynthetic pathways of rice labdane-related diterpenoid phytoalexins are shown in Figure 3 below.

## 4. Molecular Regulation of Labdane-Related Diterpenoid Phytoalexin Biosynthesis in Rice

The biosynthesis of labdane-related diterpenoid phytoalexins in rice embodies a complex molecular machinery, where a diverse array of genes and regulatory factors intertwine intricately to orchestrate sophisticated biochemical reactions. This intricate process not only underscores the profound complexity inherent in plant defense mechanisms but also emphasizes the delicate interplay and intricate interconnection between genetic determinants and environmental cues.

Among the transcription factors implicated in these processes, bZIP, bHLH, and WRKY transcription factors stand out as key regulators. These transcription factors, functioning as molecular switches, bind to specific DNA sequences in the promoter regions of biosynthase genes and modulate their expression levels in response to various stimuli. By doing so, they orchestrate the temporal and spatial expression patterns of biosynthetic enzymes, ensuring the precise and timely production of labdane-related diterpenoid phytoalexins in rice. The molecular regulatory factors of rice labdane-related diterpenoid phytoalexins biosynthesis are shown in Table 1.

### 4.1. bZIP Transcription Factors

Okada et al. reported that a bZIP transcription factor, OsTGAP1, induced by chitin oligosaccharide elicitors, plays a crucial role in the accumulation of monolignols by binding to the *OsKLS4* promoter [83]. Further studies indicated that *OsKSL4*, *OsKSL7*, and *OsDXS3* expression in the *OsTGAP1*-overexpressing lines was upregulated, and the expression levels of *OsCPS4*, *OsKSL4*, *CYP99A2*, *CYP99A3*, and *OsMAS* were nearly absent in *OsTGAP1* knockout mutant plants after induction treatment, resulting in the absence of momilactones accumulation in these mutant plants. The findings indicated that OsTGAP1 emerges as a key regulator in the biosynthesis of diterpenoid phytoalexins, exerting transcriptional control over both the genes directly involved in phytoalexin production and the upstream MEP pathway gene that facilitates the supply of geranylgeranyl diphosphate (GGPP) after elicitor recognition [83].

To deepen their understanding of OsTGAP1’s role in regulating labdane-related diterpenoid phytoalexins production, Miyamoto’s team screened for OsTGAP1-interacting proteins through yeast two-hybrid. Among candidates, OsbZIP79, a TGA factor, was studied for its physical interaction with OsTGAP1 and potential involvement in phytoalexin regulation. However, in stark contrast to the functional role of OsTGAP1, OsbZIP79 distinctly exhibits an inhibitory influence on the expression of genes that are integral to the biosynthesis of diterpenoid phytoalexins. In plants overexpressing OsbZIP79, the transcriptional levels of key diterpenoid biosynthetic enzymes, such as *OsCPS4*, *OsCPS2*, *OsKSL4*, *OsKSL7*, *CYP99A2*, *CYP99A3*, *CYP76M7*, *CYP76M8*, and *OsMAS*, are significantly downregulated, ultimately resulting in a reduced accumulation of momilactones and phytocassanes. These findings suggested OsbZIP79 acts as a negative regulator of labdane-related diterpenoid phytoalexins production in rice cells stimulated by chitin oligosaccharide elicitors, though the specific conditions for its cooperation with OsTGAP1 remain to be elucidated [84].

It is worth noting that both the expression of *OsTGAP1* and *OsbZIP79* could be stimulated by chitin oligosaccharide elicitor to regulate labdane-related diterpenoid phytoalexins biosynthesis [83,84]. Chitin oligosaccharide elicitors are recognized for their ability to trigger defense responses in plants [93,94]. In rice plants, their stimulation of *OsTGAP1* and *OsbZIP79* underscores an essential component of the plant’s innate immune system by regulating the biosynthesis of labdane-related diterpenoid phytoalexins [83,84]. Further research into the precise mechanisms and interactions of these transcription factors will provide valuable insights into the complex regulatory networks underlying plant defense.

### 4.2. bHLH Transcription Factors

Yamamura and his team have identified the bHLH transcription factor OsbHLH025, which has been named the diterpenoid phytoalexin factor (DPF) [85]. Notably, OsbHLH025 exhibits a remarkable responsiveness to various stimuli, including infection by the rice blast fungus, exposure to copper chloride, and ultraviolet light, all of which induce its expression in rice leaves and panicles [85]. 

Further research has confirmed that OsbHLH025 can directly bind to N-Box motifs present on the promoters of the key biosynthetic genes *OsCPS2* and *CYP99A2*, exerting direct regulatory control over the biosynthesis of momilactones and phytocassanes. As a result, the accumulation of momilactones and phytocassanes is significantly enhanced in *OsbHLH025*-overexpressed rice plants. Conversely, when *OsbHLH025* expression is knocked down, the accumulation of these compounds decreases correspondingly, further revealing the essential function of OsbHLH025 in regulating the biosynthesis of momilactones and phytocassanes [85]. 

It is intriguing to observe that rice plants overexpressing *OsbHLH025* (*OsbHLH025*-OX) exhibit increased accumulation of diterpenoids (DPs) even in the absence of stress treatments [85]. The phenomenon of disease-like symptoms displayed by these *OsbHLH025*-OX plants with elevated DP levels poses a challenging question to unravel. Further research is needed to fully understand the molecular mechanisms underlying the cytotoxicity of high DP levels and to develop strategies to harness the potential of these compounds for crop protection while minimizing adverse effects on plant health.

Liu and her team identified and confirmed that a bHLH transcription factor, OsbHLH026, serves as a key regulator in rice through a comprehensive approach encompassing co-expression analysis, expression quantitative trait locus (eQTL) analysis, and linkage mapping [86]. In order to study the mechanism of OsbHLH026 regulating the biosynthesis of phytoalexins, they demonstrated that OsbHLH026 protein could bind to the promoter region of *OsCPS2* and *OsKSL6* and activate these genes [86]. In the *syn*-CPP pathway, oryzalexin S (**20**), momilactones A (**1**) and B (**2**), and their intermediates *syn*-stemar-13-ene and *syn*-pimara-7,15-diene were significantly upregulated in the *OsbHLH026*-OX line compared with the wild type, whereas in the *osbhlh026*-*cri* line, only oryzalexin S (**20**) was markedly reduced, with little change observed for the others. Additionally, in the *ent*-CPP pathway, phytocassanes C (**9**), D (**10**), E (**11**), and oryzalexins C (**16**), F (**19**) were detected, with similar upregulation trends in the *OsbHLH026*-OX line as observed in the previous compounds. However, in the osbhlh026-cri line, significant decreases were observed specifically in phytocassanes D (**10**) and E (**11**) [86]. Overall, these experiments revealed that bHLH026 has a significant effect on enhancing disease resistance in rice plants, underscoring its importance in improving the overall health and resilience of the crop.

### 4.3. WRKY Transcription Factors

Akagi et al. investigated the defense-priming mechanism of benzothiadiazole (BTH) and the function of OsWRKY45 by analyzing the expression of diterpenoid phytoalexin (DP) biosynthetic genes during the interaction between rice and *Magnaporthe oryzae* [87]. They found that while BTH treatment alone slightly upregulated these genes, *M. oryzae* infection rapidly induced their expression in a WRKY45-dependent manner, indicating that BTH primes DP biosynthetic genes through WRKY45. Similarly, rice overexpressing *OsWRKY45* (*OsWRKY45*-OX) showed rapid induction of DP biosynthetic genes upon *M. oryzae* infection, including five genes involved in momilactone biosynthesis (*OsCPS4*, *OsKSL4*, *OsCYP99A2*, *CYP99A3*, and *OsMAS*), as well as those encoding enzymes crucial for phytocassane biosynthesis (*OsCPS2*, *OsKSL7*, and *CYP71Z7*) and oryzalexin biosynthesis (*OsKSL10*). These transcriptional reprogramming resulted in the accumulation of DPs in both *OsWRKY45*-OX and BTH-pretreated rice after infection. In this study, they demonstrated that the SA/CK synergy, mediated by WRKY45, regulates DP biosynthetic genes. Furthermore, they propose that CK acts as a mediator of *M. oryzae* infection signals, triggering the induction of DP biosynthetic genes in BTH-primed plants [87].

The research conducted by Chujo et al. has confirmed that the phosphorylation of the WRKY transcription factor OsWRKY53, facilitated by the OSMKK4-OSMPK3/OsMPK6 signaling cascade, results in a positive regulation of the expression of key enzyme genes, including *OsCPS4*, *OsMAS*, *CYP99A2*, and *CYP99A3* [78]. As a result, the content of momilactones and phytocassanes in rice plants increases, significantly enhancing their defense capabilities [88].

Wang’s research team precisely identified OsWRKY10 as an essential transcription factor that orchestrates the crucial upregulation of diterpenoid phytoalexin biosynthesis in rice. Notably, knockout of *OsWRKY10* dramatically enhanced the susceptibility of rice mutants to the *Magnaporthe oryzae*, underscoring its indispensable function in the defense arsenal of rice. Conversely, the amplification of *OsWRKY10* expression in transgenic rice lines robustly strengthened their resistance to rice blast, establishing a clear positive correlation. They found that OsWRKY10 could directly bind to W-boxes and W-box-like *cis*-elements (WLEs) present on the promoter regions of key biosynthetic enzyme genes, such as *OsKSL4*, *OsKSL7*, *OsKSL10*, and *CYP99A3* [89]. This direct interaction underscores the fundamental role of OsWRKY10 in modulating the biosynthesis of labdane-related diterpenoid phytoalexins in rice, thereby reinforcing rice’s natural defenses against pathogenic assaults.

Their findings reveal an intriguing phenomenon that knockout of *OsWRKY10* did not completely abolish the induction of biosynthesis genes and accumulation of rice diterpenoid phytoalexins in response to blast fungus infection or CuCl_2_ treatment [89]. This redundancy suggests that OsWRKY10, while playing a significant role, is not the sole or exclusive TF responsible for regulating the expression of genes associated with diterpenoid phytoalexin biosynthesis. Rather, there are likely other TFs that possess overlapping or complementary functions, enabling them to partially or fully compensate for the loss of OsWRKY10 activity. This highlights the robustness and flexibility of plant regulatory networks, which often employ multiple layers of redundancy to ensure resilience against perturbations. Future research should aim to unravel the full complement of TFs involved in diterpenoid phytoalexin biosynthesis, as well as their interactions and regulatory hierarchies, to gain a deeper understanding of plant stress responses and potential avenues for crop improvement.

Compared with the aforementioned WRKY transcription factors, OsWRKY76 exhibits a notable transcriptional inhibitory effect on the biosynthetic pathway of labdane-related diterpenoid phytoalexins in rice [90]. Five genes for momilactones production (*OsCPS4*, *OsKSL4*, *CYP99A2*, *CYP99A3*, and *OsMAS*), along with the three genes central to phytocassanes biosynthesis (*OsCPS2*, *OsKSL7*, and *CYP71Z7*), were downregulated significantly in the *OsWRKY76* overexpression rice plants. Consequently, this suppression diminishes the ability of the plants to produce labdane-related diterpenoid phytoalexins, ultimately heightening their vulnerability to rice blast fungus infections [90]. However, overexpression of *OsWRKY76* leads to the upregulation of abiotic stress-related genes, thereby enhancing the cold tolerance of rice [90].

### 4.4. Other Factors

Previous reports have confirmed that the plant hormone jasmonic acid (JA) is also one of the factors regulating the biosynthesis of labdane-related diterpenoid phytoalexins in rice. Mutations disrupting JA signaling lead to reduced accumulation of labdane-related diterpenoid phytoalexins upon *M. oryzae* infection, enhancing susceptibility to this pathogen [91]. Nevertheless, JA-independent mechanisms regulating DP biosynthesis are also present, as evidenced by JA-deficient mutants producing normal levels of phytocassanes despite reduced momilactones in response to *M. oryzae* [91]. Analogously, an *Osjar1-2* insertion mutant exhibits comparable labdane-related diterpenoid phytoalexins production to wild type under Cu^2+^ exposure or *M. oryzae* infection [92].

**Figure 4:**
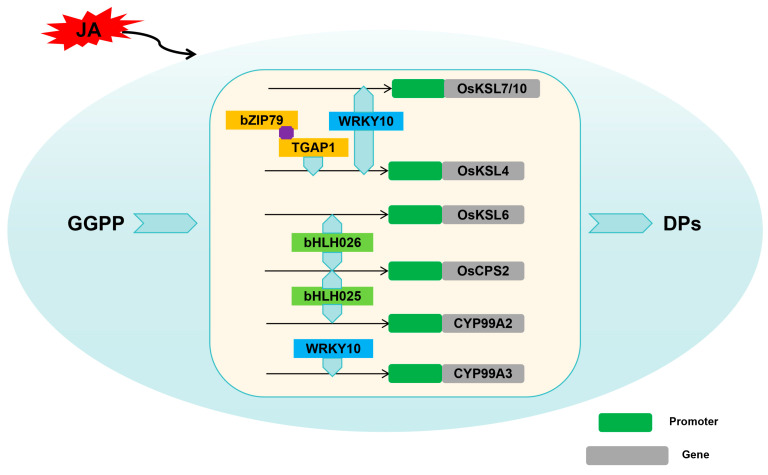
Molecular regulation of rice labdane-related diterpenoid phytoalexins.

In conclusion, studies presented in this passage provide important insights into the roles of these transcription factors in regulating the biosynthesis of labdane-related diterpenoid phytoalexins in rice (Figure 4). However, much work remains to be carried out to fully understand the signaling pathways and transcriptional networks that are regulated by this transcription factor. Future research in this area holds great promise for improving crop resilience and productivity in the face of environmental challenges.

## 5. Genetic Manipulation to Enhance Crop Resistance by Increasing the Production of Diterpenoid Phytoalexins

Currently, there are relatively few reports on attempts to enhance crop resistance by manipulating the expression of genes in the biosynthetic pathway of diterpenoid phytoalexins.

Li et al. confirmed that ectopic expression of the Harpin protein 1 gene (*hrf1*) enhances the resistance of transgenic rice plants to bacterial blight. They discovered that genes related to the biosynthesis of labdane-type diterpenoid antibiotics, specifically *OsCPS2*, *OsCPS4*, *OsKSL4*, *OsKSL7*, *OsKSL8,* and *OsKSL10* in the rice plants transformed with *hrf1*, were significantly activated. This resulted in the rapid and continuous accumulation of labdane-type diterpenoid phytoalexins in these plants, which subsequently significantly inhibited the growth of *Xanthomonas oryzae* strain PXO79 [95].

Lu et al. demonstrate that knocking out or knocking down the *OsCPS4* gene decreases susceptibility to the bacterial leaf blight pathogen *Xanthomonas oryzae*, while manipulating the expression of *OsCPS2*, which encodes the *ent*-CPP synthase, could alter susceptibility to both *M. oryzae* and *X. oryzae* [96].

*CYP71Z18* is homologous to rice *CYP71Z6* and *CYP71Z67*, which has been demonstrated to catalyze the formation of phytoalexins, including zealexin A1, sesquiterpenoid phytoalexin, and diterpenoid phytoalexin dolabralexin in maize [97]. To explore the function of CYP71Z18 in rice, Shen and his team created transgenic rice plants that overexpressed *CYP71Z18*. They discovered that the heterologous expression of *CYP71Z18* led to the accumulation of several new diterpenoid compounds in the transgenic rice. Furthermore, compared with wild-type rice plants, the transgenic rice with overexpressed *CYP71Z18* exhibited stronger resistance to *M. oryzae* infection [98].

## 6. Conclusions, Challenges, and Prospects

### 6.1. Conclusions

This article reviews the research progress in the past few decades on the biological activities, biosynthetic pathways, and molecular regulation of labdane-type diterpenoid phytoalexins in rice, aiming to provide insights for future crop resistance breeding efforts.

Rice labdane-type diterpenoid phytoalexins, including momilactones, phytocassanes, and oryzalexins, constitute an important component of rice’s defense arsenal against a variety of biotic and abiotic stresses. These naturally occurring plant metabolites exhibit diverse biological activities that contribute to the overall resilience of rice plants. Specifically, rice labdane-type diterpenoid phytoalexins display antifungal, allelopathic, and stress-responsive properties, highlighting their crucial role in safeguarding rice crop health and productivity. Their biosynthesis in rice plants primarily initiates with GGPP as the starting compound, constituting a highly orchestrated process that necessitates the intricate interplay and precise regulation of countless enzymes. In this process, some key transcription factors, such as the bZIP transcription factor, bHLH transcription factor, and WRKY transcription factor, play important regulatory roles. They can bind to the promoters of key enzyme genes in the biosynthesis of labdane-type diterpenoid phytoalexins, regulate the expression of these genes, and affect the synthesis and accumulation of these labdane-type diterpenoid phytoalexins.

Further investigation into the biosynthesis and regulation of these compounds holds promise for the development of novel strategies to enhance crop resistance against pathogens and environmental stresses. Therefore, understanding the biosynthesis and regulation of these compounds can facilitate the cultivation of new, eco-friendly crop varieties, thereby reducing the need for pesticides and herbicides, and promoting sustainable agricultural development.

### 6.2. Challenges

Given their crucial roles in augmenting plants’ natural resilience against pests and diseases, phytoalexins have emerged as prime candidates for enhancing agricultural pest and disease defense via plant genetic engineering. By strategically and precisely integrating genes encoding these phytoalexins into crop genomes, a promising pathway is paved for imparting customized and highly specific resistance mechanisms, thereby effectively protecting plants from specific pests and diseases.

However, despite the successful identification of some key enzymes and genes, the complexity of the entire metabolic network, including the integrity of its components and the dynamic interactions of regulatory mechanisms, remains to be fully elucidated. Moreover, the intricate web of interactions among genes within gene clusters poses a significant challenge. This complexity is compounded by the existence of redundant pathways and sophisticated regulatory mechanisms, which obscure the distinct functions of individual genes. Furthermore, the variations observed in gene expression patterns across different crop varieties introduce an additional layer of complexity to the research endeavor, which necessitates a nuanced and multifaceted approach to fully grasp the underlying biology.

### 6.3. Prospects

In the future, it is imperative to initially harness systematic biology approaches to meticulously unravel the intricate biosynthetic and regulatory networks governing these defense compounds such as momilactones in rice. This will provide a robust theoretical foundation for precision breeding and biosynthesis endeavors. Following this, synthetic biology techniques can be leveraged to manipulate the rice genome, either by overexpressing or fine-tuning the expression of genes associated with labdane-type diterpenoid phytoalexin biosynthesis, thereby enhancing rice’s defenses against diseases and stresses. With the continuous development of CRISPR-Cas9 and other gene editing technologies, the stress resistance potential of labdane-type diterpenoid phytoalexin could be further explored and utilized through precise editing of related genes in rice. 

**Figure 5:**
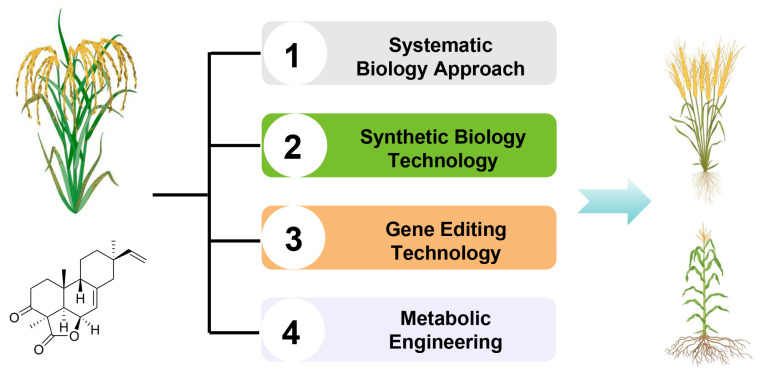
Future research strategies of rice labdane-related diterpenoid phytoalexins.

Furthermore, metabolic engineering strategies can be employed to refine the biosynthetic pathways of these defense compounds in rice, optimizing their production and efficacy. This encompasses the introduction of exogenous genes and the modification of endogenous gene expression regulatory elements to precisely control the biosynthetic pathways. In addition, the research of diterpenoids can be extended to other crops, and these favorable genes can be introduced into other crops through gene editing or transgenic technology to improve their overall stress resistance. This not only helps to reduce the use of pesticides and reduce environmental pollution but also improves the yield and quality of crops (Figure 5).

The implementation of these cutting-edge strategies promises to unlock novel insights and methodologies that will revolutionize rice disease resistance breeding and contribute to agricultural sustainability. By harnessing the full potential of diterpenoid phytoalexins, we can pave the way for more resilient and productive crop systems. In actual agricultural production, promoting the planting of these new varieties of rice with strong stress resistance can reduce the use of pesticides and fertilizers, reduce production costs, and improve the yield and quality of rice. In addition, these new varieties can also enhance the ability of rice to adapt to extreme climatic conditions and ensure the stability of food production.

## Figures and Tables

**Table 1:** Molecular regulatory factors of rice labdane-related diterpenoid phytoalexins biosynthesis.

Category	Factors	Regulation Mechanism	Reference
bZIP Transcription Factor	OsTGAP1	Upon induction by chitin oligosaccharide elicitors, OsTGAP1 binds to the *OsKLS4* promoter, positively regulating the expression of genes including *OsCPS4*, *OsKSL4*, *CYP99A2*, *CYP99A3*, and *OsMAS*.	[83]
	OsbZIP79	The expression of *OsCPS4*, *OsCPS2*, *OsKSL4*, *OsKSL7*, *CYP99A2/3*, *CYP76M7*, *CYP76M8*, and *OsMAS* is negatively regulated upon stimulation by chitin oligosaccharide elicitors	[84]
bHLH Transcription Factors	OsbHLH025	OsbHLH025 directly interact with the promoters of the key biosynthetic genes *OsCPS2* and *CYP99A2* to regulate accumulation of momilactones and phytocassanes	[85]
	OsbHLH026	OsbHLH026 protein binds to the promoter regions of OsCPS2 and OsKSL6 and activate the expression of these genes.	[86]
WRKY Transcription Factors	OsWRKY45	Genes encoding enzymes involved in momilactone biosynthesis (*OsCPS4*, *OsKSL4*, *CYP99A2*, *CYP99A3*, and OsMAS), phytocassane biosynthesis (*OsCPS2*, *OsKSL7*, and *CYP71Z7*) and oryzalexin biosynthesis (*OsKSL10*) were upregulated in both *OsWRKY45*-OX rice lines	[87]
	OsWRKY53	Phosphorylation mediated by the OSMKK4-OSMPK3/OsMPK6 signaling cascade mediated *OsWRKY53* positively regulates the expression of key enzyme genes, including *OsCPS4*, *OsMAS*, *CYP99A2* and *CYP99A3*.	[88]
	OsWRKY10	OsWRKY10 protein directly binds to W-boxes and W-box-like cis-elements (WLEs) present in the promoter regions of key biosynthetic enzyme genes, including *OsKSL4*, *OsKSL7*, *OsKSL10*, and *CYP99A3*	[89]
	OsWRKY76	Five genes for momilactones production (*OsCPS4*, *OsKSL4*, *CYP99A2*, *CYP99A3*, and *OsMAS*), along with the three genes central to phytocassanes biosynthesis (*OsCPS2*, *OsKSL7*, and *CYP71Z7*) were down-regulated in *OsWRKY76* overexpressing rice plants.	[90]
Plant Hormone	JA	Mutations disrupting JA signaling lead to reduced accumulation of labdane-related diterpenoid phytoalexins upon *M. oryzae* infection	[91,92]

## Abbreviations

EC_50_	Half-maximal effective concentration
IC_50_	Half-maximal inhibitory concentration
CYPs	Cytochrome P450 enzymes
SDR	Short-chain dehydrogenase reductase
DiTPSs	Diterpene synthases
CPSs	Copalyl diphosphate synthases
KSL	*ent*-kaurene synthase like
GGPP	Geranylgeranyl diphosphate
*ent*-CPP	*ent*-copalyl diphosphate
*syn*-CPP	*syn*-copalyl diphosphate
MEP	2-C-methyl-D-erythritol 4-phosphate
MVA	Mevalonate
DMAPP	Dimethylallyl diphosphate
IPP	Isopentenyl diphosphate
GA-3P	Glyceraldehyde 3-phosphate
CoA	Coenzyme A
DPs	Diterpenoid phytoalexins
DPF	Diterpenoid phytoalexin factor
BTH	Benzothiadiazole
JA	Jasmonic acid
SA	Salicylic acid
CK	Cytokinin
UV	Ultraviolet radiation
